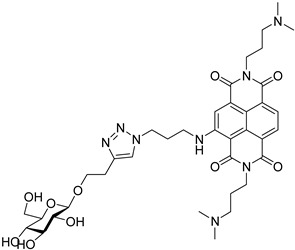# Naphthalene Diimides as Multimodal G-Quadruplex-Selective Ligands

**DOI:** 10.3390/molecules24030426

**Published:** 2019-01-24

**Authors:** Valentina Pirota, Matteo Nadai, Filippo Doria, Sara N. Richter

**Affiliations:** 1 Department of Chemistry, University of Pavia, Viale Taramelli 10, 27100 Pavia, Italy; filippo.doria@unipv.it; 2 Department of Molecular Medicine, University of Padua, via A. Gabelli 63, 35121 Padua, Italy

**Keywords:** G-quadruplex, naphthalene diimide, telomeres, telomerase, oncogene promoters, ligands, anticancer activity, viruses, parasites, binding modes

## Abstract

G-quadruplexes are four-stranded nucleic acids structures that can form in guanine-rich sequences. Following the observation that G-quadruplexes are particularly abundant in genomic regions related to cancer, such as telomeres and oncogenes promoters, several G-quadruplex-binding molecules have been developed for therapeutic purposes. Among them, naphthalene diimide derivatives have reported versatility, consistent selectivity and high affinity toward the G-quadruplex structures. In this review, we present the chemical features, synthesis and peculiar optoelectronic properties (absorption, emission, redox) that make naphtalene diimides so versatile for biomedical applications. We present the latest developments on naphthalene diimides as G-quadruplex ligands, focusing on their ability to bind G-quadruplexes at telomeres and oncogene promoters with consequent anticancer activity. Their different binding modes (reversible versus irreversible/covalent) towards G-quadruplexes and their additional use as antimicrobial agents are also presented and discussed.

## 1. Introduction

**Figure 1:**
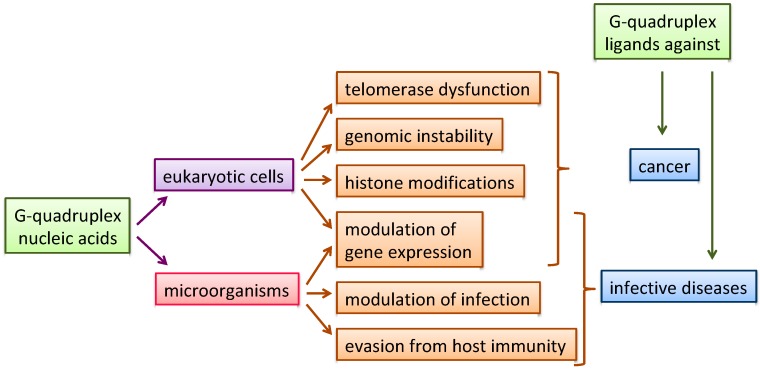
Schematic representation of G4-modulated organisms, biological processes and fields of therapeutic applications.

Nucleic acids (NAs) are key targets in medicinal chemistry; in particular, non-canonical NA structures, including G-quadruplex structures (G4s), have been proposed to be involved in genomic instability [1,2], telomerase dysfunction [3], histone modifications [4], modulation of gene expression [5,6,7,8] and viral transcription [9,10], providing possible targets in a number of different diseases. Moreover, G4 structures, from the earlier work of Hardin et al. [11] to the most recent evidence highlighted by Chowdhury [12], are arising as promising regulators of epigenetic modifications [13,14,15]. As a natural consequence, selective recognition and stabilization of G4s by small molecules is emerging as a new general strategy for innovative therapeutics (Figure 1) [16,17,18,19,20,21,22,23].

In principle, any repetitive guanine-rich region may be able to fold into polymorphous four-NA-strand G4s. Four guanine bases can associate through Hoogsteen-type hydrogen bonds to form the G-quartet, a square planar structure. Two or more G-quartets can stack on top of each other, forming the G4, which is further stabilized by cations (Na^+^ or K^+^) located between the G-quartets [24]. 

**Figure 2:**
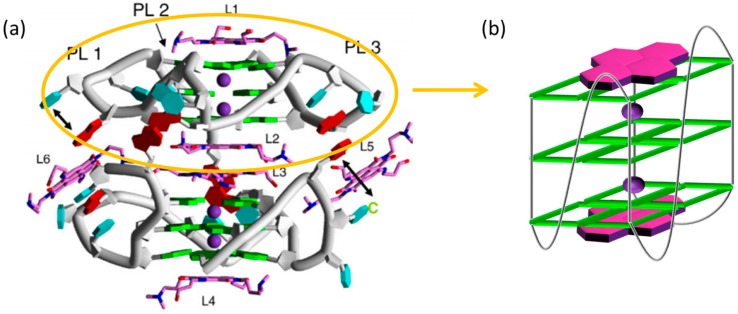
(**a**) The crystal structure of the 23-mer intramolecular G4 d[TAGGG(TTAGGG)_3_] complexed with tetra-substituted naphthalene diimide ligands (Figure taken from [25]); (**b**) model of the NDI-G4 binding through π-stacking interactions.

Based on this peculiar common structure, G4-interacting compounds can be rationally designed according to the two following criteria: (1) Extended aromatic and electron poor surface (π-stacking interaction) and (2) cationic side chains (electrostatic attraction). Many small molecules that bind G4s have been developed, with several of them showing biological activity. Although there is still a long way to go in the development of drugs that selectively target one specific G4 structure, promising lead compounds that act in the cell have been presented. Among them, naphthalene diimides (NDIs) are a class of small molecules able to recognize, induce and stabilize G4 structures with high affinity, through π-stacking interactions (Figure 2).

Herein we report an overview on the NDI properties and their use as G4 ligands, with a particular focus on their anticancer achievements.

## 2. General Features, Synthesis and Applications of Naphthalene Diimides

**Figure 3:**
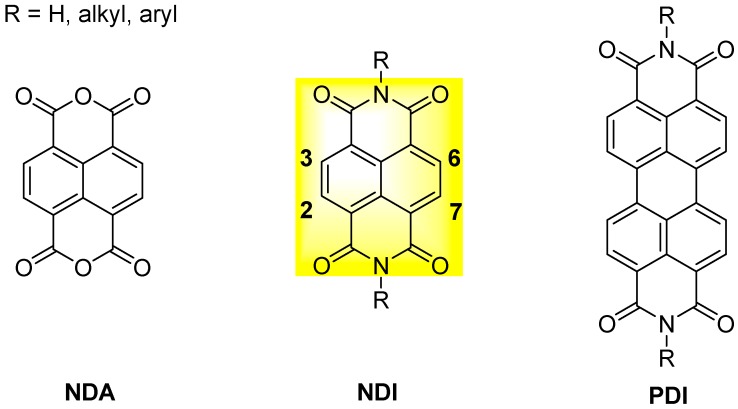
General structure of naphthalene dianhydride (NDA), naphthalene diimide (NDI) and perylene diimide (PDI).

1,4,5,8-Naphthalenediimides (NDIs) are perylene diimides (PDIs) smaller analogues (Figure 3) [26] composed of a single naphthalene core that bears two electron-withdrawing imide groups; the two nitrogen atoms can be substituted with a variety of alkyl or aryl chains. Unlike PDIs, which display outstanding absorption properties and are therefore of high significance as industrial pigments, naphthalene diimides without substituents at the aromatic core are colourless and therefore of no use as pigments. Nevertheless, NDIs are the most important PDI homologues. Addition of a single substituent at the aromatic core turns the NDI into a versatile large π-planar chromophore with interesting optoelectronic properties. Although the first reports on core-substituted naphthalene diimides (cNDIs) date back to 80 years ago, consistent research only began in the XXI century, when the synthetic route was improved and, more importantly, interesting chemical-physical properties were discovered. Due to their electron-deficient π-system, NDIs are now a class of compounds extensively studied in the field of supramolecular and materials chemistry. NDIs possess high electron affinity, good charge carrier mobility, and excellent thermal and oxidative stability. Thanks to their planar aromatic core, NDIs exhibit aromatic π-stacking and van der Waals interactions: the electron density on most aromatic rings creates a quadrupole moment with a partial negative charge above and below the plane and a partial positive charge around the periphery. This set of features makes NDIs promising candidates for a variety of applications in the field of material sciences; for example, they have been employed as n-semiconductors, photovoltaic devices, flexible displays and molecular tweezers [26,27,28,29,30,31]. Moreover, functionalization through core-substitution (2,6- or 2,3,6,7- to the core, Figure 3) produces cNDI analogues with modular absorption and fluorescence properties, positioning them among the most versatile and fascinating aromatic dyes [32].

**Figure 4:**
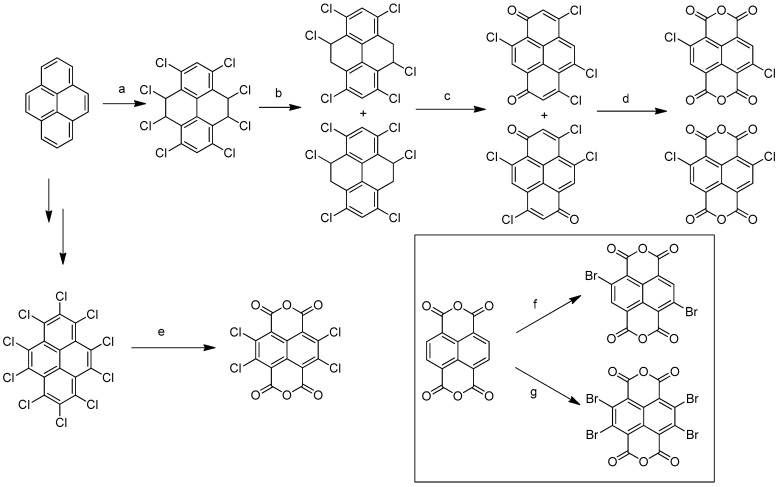
General synthetic route to access chloro and bromo cNDIs. (**a**) Cl_2_ (**g**), y = 36–38%; (**b**) KOH/EtOH, y = 96–97%; (**c**) HNO_3_ y = 32–45%; (**d**) HNO_3_/H_2_SO_4_, y = 45–50%; (**e**) oleum, 100 °C, y = 25%; (**f**) DBI, oleum, RT, y = 80%; (**g**) excess DBI, oleum, RT, y = 93%.

**Figure 5:**
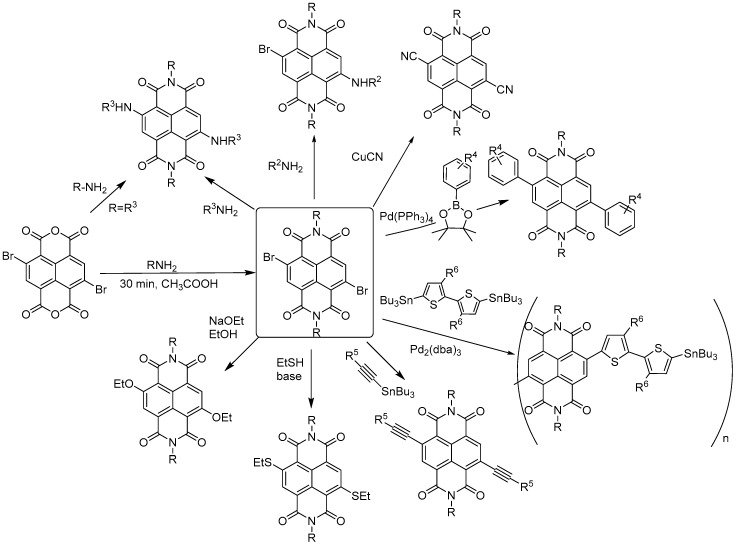
Synthetic routes to core di-functionalization.

The synthesis of cNDIs starts from the halogenated naphthalene dianhydride (NDA) (Figure 4) prepared from pyrene. For the first half of the 20th century, the Vollmann’s method, the traditional synthetic route, employed a harsh oxidative halogenation to retrieve the di-chlorinated NDI in four steps [33]. Despite the fact that this method made halogenated NDI derivatives accessible for many years, the intense application prospects of cNDIs imposed the development of an alternative, more direct, and easier synthetic approach. Thalacker and Wurthner reported the first direct bromination of the commercially available NDA with one equivalent of dibromoisocyanuric acid (DBI) to afford the 2,6-dibromo dianhydride (Figure 4) [33]. The use of an excess of halogenating agent led to the 2,3,6,7-tetrabromo dianhydride. Starting from these two key intermediates, a multitude of chemical modifications were performed, as recently reviewed by Würthner and co-workers (Figure 5) [32].

Four-site functionalization with the one substituent can be carried out in a single step in polar solvents at high temperature [34]. This set of conditions is particularly favourable for reductive dehalogenation [35]. Since the primary amine is an oxidizing agent, preservation of the four bromine atoms is difficult, unless bulky amines are used as reagents. By modulating the reaction conditions, it is possible to prevent reductive dehalogenation and introduce structural diversity to the naphthalene diimide core. The general protocol starts with the imidation reaction that leads to symmetric diimide substituents. It is then possible to obtain the orthogonal core-functionalization with stepwise nucleophilic substitutions [33]. The first step is carried out in acetic acid, ensuring optimal pH conditions for the catalytic protonation of the carbonyl and amine, in order to reduce nucleophilicity of the latter to a level that prevents aromatic substitutions [36]. Both chloro- and bromo-derivatives allow further nucleophilic aromatic substitutions that can be achieved through a non-catalysed process with classical nucleophiles, such as amines, alcohols, thiols, and cyanides. Alternatively, organometallic chemistry can be used, e.g., Suzuki–Miyaura coupling with boronate esters to attach phenyl groups or Sonogashira/Stille couplings to introduce alkynes and heterocycles (Figure 5) [27,32].

**Figure 6:**
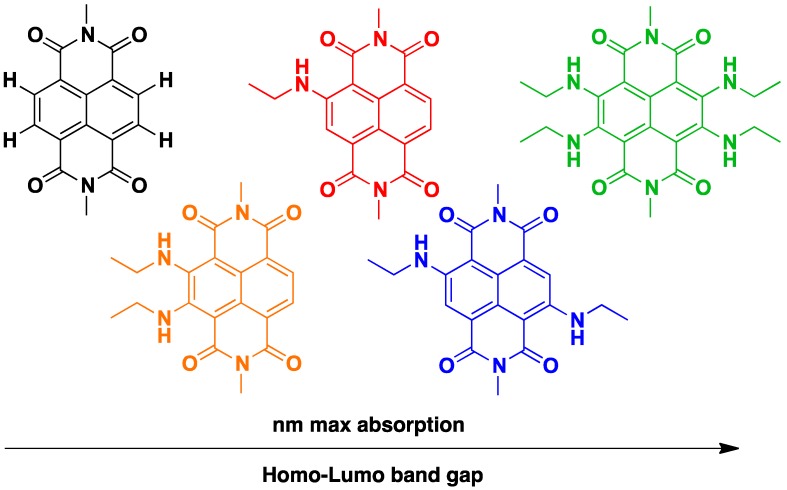
A rainbow collection of cNDIs.

The easily tuneable synthesis of the cNDIs allows the conjugation of the NDI core to different heteroatoms. In particular, positions 2, 3, 6 and 7 of the cNDI aromatic core (Figure 3) can bear one or more substituents with different-extent electro-donor effect on the π system [32]. This in turn modulates the cNDI photophysical properties providing, for example, bright colouring and high fluorescence, both over a wide range of wavelengths [37]. The ability of cNDIs to change colour and redox properties without modifying their global features is one of their most attractive features. The typical colours of cNDIs originate from a charge-transfer band that, depending on the substituent, moves along the entire visible spectra. This property is related to the HOMO-LUMO “bandgap” (Figure 6) [38]. 

Besides their use as pigments and dyes, NDIs are also widely employed as main planar components of bio-polymers and supramolecular nanostructures. The existence of delocalized radical anions in the NDI π-stacks confers the ability to work as efficient electron transporters to these molecules. For this reason, cNDIs have been identified as a class of the few existing air-stable n-semiconductors used to generate high-performance organic thin-film transistors (OTFTs) [39,40]. The known spectroscopic properties matched to the ability to transport electrons make cNDIs the ideal systems for the photoinduced electron transport, supporting construction of photovoltaic cells and artificial photosynthesis. The photophysical properties of cNDIs are also well suited for sensing applications. In the literature, there are many examples of fluorescence sensor of different types, such as anion sensors, fluorescent chemosensors for zinc ions and pH sensors [41]. Their fluorescence associated with the flatness of the chromophore makes cNDIs useful for biomolecular recognition. Moreover, some tetrasubstituted NDIs can be used as photosensitizers for photodynamic therapy (PDT) [42,43]. These molecules exhibit large inter-system crossing quantum yields, whereby the generated triplet states are efficiently quenched by molecular oxygen to produce singlet molecular oxygen with high quantum yields. 

In the field of biomedicine, cNDIs are used as scaffolds for G4-selective ligands. Their chemical accessibility, large planar electron-poor surface and the presence of protonable side chains are the most relevant features exploited for G4 binding. Thanks to the π acidity, a peculiar characteristic of the electron-deficient aromatic systems, the NDI core is prone to interact by π-π stacking with an electron-rich partner, like the G-quartet. In addition, the side chain substituents, which are often protected tertiary amines or hydroxyl groups, can form hydrogen bonds with the phosphate groups in the G4 grooves, thereby stabilizing the G4-NDI complex. 

These properties have not only been investigated from the therapeutic point of view: Over the last decade, the NDI core has also become important in the field of diagnostics. By combining the cNDI outstanding fluorescent properties with its ability to interact and recognize the G4 structure, a wide range of selective chemosensors was generated [44,45,46,47,48,49]. Although most of the red-NIR G4 binders exhibit quenching upon binding, the cNDIs belonging to this class of sensors are able to selectively switch on their fluorescence properties upon G4 recognition. This selective response has made possible the use of one of these sensors in live cells, where excellent co-localization with an anti-G4-antibody (1H6) was obtained [50].

## 3. NDI Derivatives That Bind to Human Telomere G-Quadruplexes and Inhibit Telomerase Activity

### 3.1. Reversible NDI Ligands 

Around 10 years ago, Neidle’s group was the first to report stabilization of telomeric G4s by tri- and tetrasubstituted cNDIs, resulting in telomerase inhibition [34]. The presented library of NDIs, bearing different aliphatic tertiary amine or hydroxyl groups on the side chains (compounds **1**–**24** in Table 1), was investigated on two different DNA sequences: the human telomeric DNA (able to fold into G4) and a self-complementary duplex DNA hairpin. In general, an increase in the number of substituents corresponded to an increase in G4 stabilization: tetra-substituted cNDIs showed great selectivity for G4 vs duplex DNA in competition experiments. Moreover, enhanced G4 selectivity was obtained reducing the protonable sites in the side chains, e.g., with a morpholine cycle or hydroxyl group (compounds **13**–**16**, **19**, **20**, **22**, Table 1). These compounds reported melting values in telomeric G4 stabilization, measured by fluorescence resonance energy transfer (FRET) analysis, comparable to those obtained with well-known G4 ligands, such as BRACO-19. However, the cNDIs showed higher potency: the same degree of G4 stabilization was reached at 0.5 µM of cNDIs compared to 2.5 µM of BRACO-19. These cNDIs were able to inhibit telomerase with EC_50_ values in a µM range (compounds **1**, **2**, **5**, **6**, **9** and **11**, Table 1), as measured by a modified telomeric repeat amplification protocol (TRAP) assay. The most potent cNDIs (compounds **2**, **3**, **5**, **11** and **12**, Table 1) arrested cell growth of MCF7 and A549 cancer cell lines with IC_50_ values of 5–20 nM, as assessed in the sulforhodamine B assay. As the corresponding IC_50_ values on a normal human fibroblast line (WI38) were typically greater than one order of magnitude, a potentially promising therapeutic index in the above cancer cell lines was indicated. The relation between G4 ligand activity at the telomere level and cellular senescence was supported by confocal microscopy experiments that showed exclusive cNDI localization in the nuclei, i.e., where telomerase acts.

The quantitative interactions of cNDIs **1-9-22** (Table 1) were further analysed on human telomeric RNA (TERRA) quadruplex using electrospray mass spectrometry methods, comparing the obtained results with BRACO-19 [51]. While, all the three cNDIs bind to the telomeric DNA with high affinity, only **22** guarantees a strong formation of the NDI/RNA-G4 complex, showing equilibrium dissociation constants of *K_d_1* = 4.0 µM and *K_d_2 =* 5.0 µM, respectively. These results were also justified by a molecular dynamics simulation [51,52].

These molecules were particularly potent in G4 binding and telomerase inhibition; therefore, the NDI scaffold became the landmark for some of the most active small molecules able to efficiently target G4s. The NDI core was subsequently modified with manifold side chains, each with fundamental characteristics, in order to improve the selective interaction with the G4 target and the transition across the nuclear membrane.

The NDI core functionalized with tetra *N*-methyl-piperazine end groups (compounds **25**–**27**, Table 1) allowed to obtain crystallographic data of the cNDI-G4 complexes, highlighting a 1:1 stoichiometry, the interaction of positive charges with the groove, and the ability to induce the parallel topology of the human intramolecular telomeric G4 [53]. These small molecules presented high short-term cytotoxicity (IC_50_ around 0.1–0.2 µM) on a number of cancer cell lines (MCF7, A549, MIA-Pa-Ca-2, PANC-1, HPAC, BxPc-3) and resulted significantly less toxic on normal fibroblasts WI38 (IC_50_ increased of 2 order of magnitude) [54]. Based on the observation that the three-carbon atom linker (compound **25**, Table 1) guaranteed the highest efficiency of cell entry, the mechanism of action of this compound was further studied. This molecule resulted in an effective stabilizer of the human telomeric G4s, able to displace the single-strand binding protein hPOT1 from telomeric ends. However, cell data suggested an additional contribution to its antitumor action: the possible simultaneous targeting of several G4s that induced a therapeutically beneficial HSP90 down-regulation. 

Two *N*-methyl-piperazine units were subsequently replaced by morpholine groups (compound **28**, Table 1) without loss of the intermolecular interaction of the cNDI side chains with the G4 grooves [55]. These cNDIs showed superior cell growth inhibition, in particular towards the pancreatic MIA PaCa-2 carcinoma cells (IC_50_ values around 10 nM), thus looking particularly promising for in vivo therapeutic applications. Unfortunately, no telomerase inhibition activity was found in this cell line at the nanomolar dosage level required to inhibit cellular proliferation. 

Considering that the interaction with the G4 grooves is fundamental to improve G4-ligand binding, Czerwinska et al. proposed NDIs carrying a dipicolylamine unit as Zn^2+^ coordinating moiety (compound **29**, Table 1) [56]. As hypothesized, the Zn^2+^ complex provided additional anchorages to the telomeric G4 through electrostatic interaction with the negative charges of the target. This ligand might be a good anticancer candidate (EC_50_ value around 5 µM), but further comparative studies will be necessary.

It has been shown that a large positive charge on one hand guarantees NDIs cellular uptake, on the other reduces the selectivity towards the target due to electrostatic interaction, which takes place also with the dsDNA. To solve this problem, Morales et al. engineered carbohydrate-cNDI conjugates (carb-cNDIs) that entered into cancer cells taking advantage of the overexpressed glucose transporter (GLUT) (compounds **30**–**32**, Table 1) [57]. In particular, while aglycone cNDI **32** was quickly uptaken by passive diffusion or facilitated transport both in cancer cell lines HT-29 and MCF-7 and in the noncancerous cell line MRC-5, the uptake of compounds **30**–**32** in HT-29 and MCF-7 cells, where the GLUT is overexpressed, was more efficient. The compounds’ transport into cancer cell depended on the type of carbohydrate present on the NDI as well as on the compound incubation time and temperature.

### 3.2. Multimodal NDI Ligands

Taking into account the excellent binding properties of NDIs towards G4s, multimodal ligands that added the ability to selectively damage the targeted G4s were developed. The idea behind this approach is activation of a chemical reactive moiety on the NDI core only upon NDI recognition and stabilization of the therapeutic target G4s, in order to minimize typical off-target reactivity. 

Freccero’s group developed hybrid structures that combine the G4-recognizing NDI core with quinone methide (QM) precursor units as activatable alkylating agents (compounds **34**–**48**, Table 2) [58,59,60]. QMs are short-lived reactive Michael acceptors that can be generated in situ by mild thermal activation (40 °C), which is compatible with physiological conditions. The study of these alkylating ligands started with bis-substituted NDIs (**34**–**36**), where the activation of the quaternary ammonium salts was obtained by mild thermal incubation at 40 °C, pH 7, for less than 12 h [58]. Despite the presence of two symmetric QM precursors (QMP), only the mono alkylated telomeric G4 target was retrieved. The best compound of the series (**35**) formed a G4-NDI covalent adduct that was experimentally visible, starting from a compound concentration of 0.25 µM; at 2 µM, maximum alkylation (15%) over total DNA concentration was obtained. Direct comparison of compounds **34** and **36**, indicated the key role of the alkyl-spacer in telomeric G4 alkylation. These compounds reported high selectivity towards G4-folded DNA vs dsDNA and a ss-scrambled-DNA unable to fold into G4.

Subsequently, tri- and tetrasubstituted cNDIs containing only one QMP unit were synthetized to improve alkylation efficiency. In this case, the QMP was tethered to the aromatic core (compounds **37**–**48**, Table 2) and two conformational flexible amines were introduced as solubilizing side chains. FRET-melting values obtained for the non-alkylating compounds (**37**–**40**) were in line with other tri- and tetrasubstituted cNDIs, with the best ligand of the series exhibiting ΔT_m_ above 20 °C at 0.8 µM [59]. Interestingly, compound **37** inhibited human melanoma SKMeI-5 cell growth at 48 h with IC_50_ of 1.7 ± 0.5 µM. The marked reduction of telomeric 3′-overhang, the decrease in the amounts of both the telomere-associated proteins TRF2 and hPOT1 and the increase in γ-H2AX expression levels clearly indicated a noticeable perturbation at the telomeric level. 

cNDI-QMPs analogues (**41**, **42**) reported modest alkylation, with the highest percentage of alkylated adduct of 12% at 50 µM of compound **41**. Formation of the alkylated adducts was detectable starting from 12 nM (compound **41**) and 50 nM (compound **42**) and only with the target telomeric G4. However, these compounds showed poor cellular permeability.

Further improvements were introduced in compounds cNDI-QMPs **43**–**48**, in which a phenol moiety was introduced on the cNDI using alkyl-amido spacers [60]. The best alkylating agents of this series were **43** and **44**, which presented alkylation starting from 30 nM compound concentration; alkylation maximum yields of 16.8% and 12.8%, respectively, were obtained at compound concentration of 10 µM. Alkylation was particularly selective for the G4 folded DNA structures and cytotoxicity towards two telomerase-positive human carcinoma cell lines (lung and colon) was 2–10 times higher in comparison with the effect observed on telomerase-negative human foreskin fibroblasts.

The above-described alkylating hybrid ligands constituted the first step towards the rational design and synthesis of new molecular devices capable of recognizing, stabilizing and selectively modifying the G4 targets with high efficiency. Freccero’s group next developed a cNDI functionalized with an intrinsically reactive oxirane unit (**49**, Table 2) [61]. Compound **49** formed adducts with G4 DNA that were stable up to 90 °C and selectively alkylated adenine versus guanine bases in the hTel G4 target. Adduct yield of 16% was obtained at the cNDI/G4 hTel ratio of 12:1, while non-significant alkylation was obtained on ss and ds DNA substrates.

In 2015, the same research group conducted further studies on a photoreactive G4 ligand (**37**, Table 2) formed by a trisubstituted cNDI dye conjugated to phenol moiety [62]. Upon green light (532 nm) laser excitation, compound **37** generated a phenoxyl radical by intramolecular photoinduced electron transfer (PeT). This carbon radical selectively reacted with thymine residues in the loops of the telomeric G4, inducing formation of 64% of covalent adduct over total DNA at compound concentration of 12.5 µM. The high adduct formation efficiency on the G4 was paralleled by hardly detectable adduct formation on a ss-DNA unable to fold into G4 and no adduct on ds-DNA (even using 200-fold compound excess). Moreover, compound **37** exhibited IC_50_ of 0.5 µM in the MCF7 breast cancer cell line under green light activation; the IC_50_ value was four times higher when the compound was maintained in the dark.

In order to verify singlet oxygen formation in PDT conditions, Freccero’s group synthetized a tetra-cationic quaternary ammonium cNDI. Compound **50** (Table 2) presented a singlet oxygen quantum yield of 0.30 upon excitation with red light [42] alongside good G4 binding abilities, similar to those of other tetrasubstituted cNDIs. Compound **50** accumulation into the nucleus of all analysed tumour cell lines (HeLa, BJEHLT and U2OS) was visualized by exploiting its intense emission band in the near-IR. Interestingly, the compound did not display cytotoxicity in the absence of red-light irradiation, a property that allows cNDI activation and hence DNA damage only when the compound is firmly linked to its therapeutic target. Reduction of cell viability up to 40% in BJEHLT and HeLa cell lines and a significant increase of cells expressing γH2AX foci (indicating DNA damage) were reported. However, the presence of four positive charges increased the general binding to nucleic acids due to electrostatic interactions. As a matter of fact, cNDI **50** was less selective towards the G4 structure, with dsDNA as an important off-target.

## 4. NDIs as G4-Mediated Downregulators of Gene Expression 

G4s have been shown to be non-randomly distributed in the human genome: besides telomeres, G4s are mainly present in gene promoters and in regions that are close to transcription start sites (TSS) [63]. Recently, a Ch-IP sequencing-based genome-wide mapping of G4s showed enrichment of G4s in cancer-related genes, with MYC having the highest G4 density [64]. Altogether these data support a role of G4 structures in the progression of cancer [65]. 

These observations led to the hypothesis that G4 induction and stabilization by small molecules could be exploited to repress gene transcription. This hypothesis was first demonstrated with the porphyrin ligand TMPyP4, which was able to downregulate MYC expression [66], paving the way for the quest of specific G4-stabilizing molecules.

### 4.1. NDI Derivatives Targeting Oncogenes

**Figure 7:**
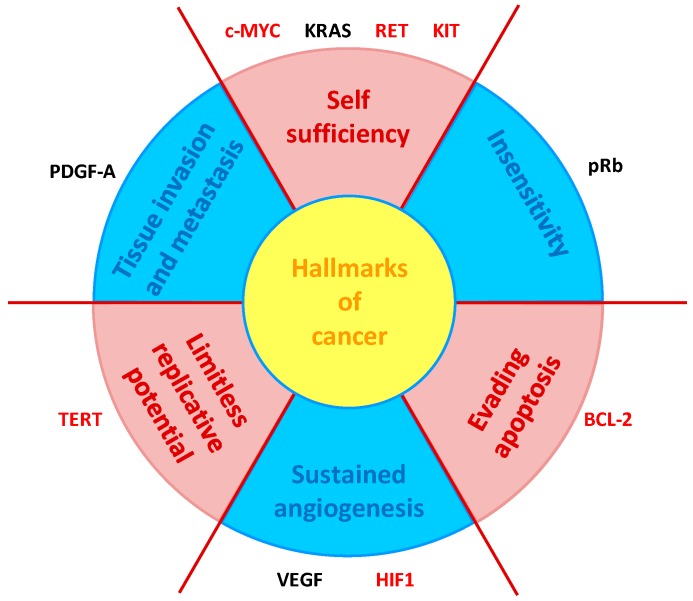
Schematic representation of the six hallmarks of cancer [67], in relation with genes containing G4s in their promoters. Gene marked in red are targeted by one or more NDIs described in this session (adapted from [68]).

Based on the observation that all processes involved in oncogenic transformation and malignancy—the so-called hallmarks of cancer [67]—are represented by one or more genes containing G4s in their core or proximal promoter (Figure 7) [68], while tumor suppressor genes promoters notably lack G4s [69], a large part of G4 ligands has been designed to target oncogene promoters. In addition, given the multifactoriality of cancer and the involvement of G4s in all its hallmarks, the typical lack of specificity of the ligand towards one single G4 can become an advantage [70].

In this session, we will describe the NDIs developed against each oncogene promoter.

Table 1, Table 2 and Table 3 represent structures of the molecules considered in this section, while in Table 4, we have summarized and compared the in vitro and in cell activity of the presented NDI compounds. However, as a note of caution in interpreting this table, we wish to mention that the correlation between stabilization in vitro and cell activity is not always straightforward due to a series of reasons: the G4 target measured in vitro may not be the sole G4 targeted in cells (as a matter of fact not all G4 in cells have been characterized, therefore some may be targeted without even being recognized as targets). In addition, in cells additional factors come into play: these are related to the cells and their inherent variability. For example, a G4 ligand could be very active in vitro and be less active in cells due to poor entry or localization properties. Poorer or better cell entry may be found in different cell lines. Different cell lines may not be expressing the relevant target, or the same cell line at different cell life stages may be not be expressing the same G4 repertoire [64]. All these factors impact on the relationship (or absence of it) between the in vitro and in vivo results. In addition, activities measured in different labs can also be quite different based on the different experimental settings, and thus a direct comparison might not be straightforward.

#### 4.1.1. KIT

The human proto-oncogene KIT encodes for a tyrosine kinase receptor that stimulates cell proliferation, differentiation and survival [71]. When mutated or overexpressed, KIT results in aberrant function and oncogenic cellular transformation leading to cellular self-sufficiency in growth signals. Activating mutations in KIT are responsible for most of the gastro-intestinal stromal tumors (GIST) and many of the current therapies that target KIT and PDGFRA proteins result in the onset of resistance. Thus, direct targeting of the KIT gene can be an alternative and fruitful approach.

The KIT promoter contains two G4-forming sequences upstream its TSS, namely KIT1 and KIT2 [72,73], whose NMR structures have been solved [74,75].

Several G4 stabilizers have been tested against KIT, including NDIs. The first report presented a tetrasubstituted cNDI (compound **11** in Table 1 and Table 3) [34] that stabilized both KIT-2 and the human telomeric sequence with similar ΔT_m_ values (ΔT_m_ 29.0 °C and 28.7 °C, respectively, Table 4): its activity was higher than that of other compounds such as BRACO-19 and TMPyP4, as assessed by FRET melting analysis [76]. Molecular modeling data of compound **11** on the human telomeric structure showed a higher affinity than the reference compound BRACO-19, paralleling the FRET melting data. Short-term antiproliferative activity showed values similar to imatinib, the reference drug for GIST treatment, in GIST cell lines (IC_50_ 1.6 µM, Table 4); moreover, the NDI was able to reduce KIT transcription by 90% and to completely inhibit KIT expression at the same dosage level required to obtain antiproliferative effect in cells. Given the potent telomerase inhibition, assessed by TRAP-LIG assay, the authors hypothesized a dual mode of action, with inhibition of both telomerase activity and expression of the KIT gene.

A series of tetrasubstituted cNDIs, compounds **25**–**27** in Table 1, previously reported as stabilizers of the telomeric G4 and telomerase inhibitors [54], were able to stabilize also the G4s in the promoter region of the KIT and HIF-1α oncogenes, and the G4 in the mRNA of BCL-2 (Table 4) [77]. Compound **25** (Table 3) was able to initiate senescence in cancer cells, probably as a consequence of telomere uncapping through G4 stabilization, and subsequent DNA damage, as demonstrated by the detection of γ-H2AX foci. Furthermore, the compound led to telomeric chromosomal fusion, resulting in genomic instability and subsequent apoptosis. The closely related molecule **27** (Table 1) was able to enter cell cytoplasm and nucleoli, as assessed by confocal microscopy; it induced an increase of telomeric associations in cells, stimulating chromosomal instability.

Macrocyclic NDIs bearing spermidine- and spermine-like side chains (compounds **51**–**54**, Table 3) [78] stabilized both the KIT2 and telomeric sequences (ΔT_m_ 33.1 °C and 26.8 °C, respectively, Table 4) in biophysical assays, but they were not able to inhibit telomerase. Computational studies confirmed that the most stabilizing molecule was also the best G4 binder and proposed the electrostatic contribution of the side chains as the driving force for the binding process. However, in cells, the antiproliferative effect was not related to G4 stabilization (the higher the ΔT_m_, the lower the antiproliferative effect, and vice versa), and lacked cell-type selectivity: this was likely due to the physicochemical properties of the molecules, or to a different mechanism of action.

NDIs functionalized with disubstituted aminoacids were synthetized and investigated as G4 binders [79]: one of them (compound **55**, Table 3) showed selective stabilization of the KIT2 sequence (ΔT_m_ 14.6 °C, Table 4) coupled with a slight destabilization of the KIT 1 sequence in vitro.

#### 4.1.2. BCL-2

BCL-2 is an antiapoptotic gene containing several G4-forming sequences in its promoter [80,81,82] and one in the 5′-UTR of its mRNA [83]. These sequences are able to fold into different intramolecular structures, having a critical role in BCL-2 gene expression regulation [84]. Over-expression of the BCL-2 protein prevents apopotosis and has been found to interfere with cancer therapeutics [85].

The tetrasubstituted cNDI derivative **28** (Table 1 and Table 3) belongs to a previously reported series of molecules that had been characterized as potent stabilizers of different G4s and antiproliferative agents in a panel of cancer cell lines, without being telomerase inhibitors [55]. Subsequently, **28** was reported to stabilize the KRAS1, KRAS2 and BCL-2 G4 sequences, with a slight selectivity for the latter (ΔT_m_ 22.5 °C, 19.8 °C and 26.4 °C, respectively). In vivo studies showed nuclear localization in tumor cells, reduction of about 80% of the tumor growth in mice, and reduction of KRAS and BCL-2 protein levels in treated tumors (Table 4) [86].

In addition, the tetrasubstituted cNDI **11** (Table 1 and Table 3), active against KIT [76] as presented above, stabilized the BCL-2 G4 [87]. The compound showed high antiproliferative activity in a GIST-imatinib resistant cell line (GIST48) in short-term experiments (IC_50_ 0.5 ± 0.05 µM, Table 4), did not affect telomerase activity in this cell line, and did not have a large effect on the KIT gene. Interestingly, this cNDI was able to strongly reduce (about 85%) the expression level of the BCL-2 protein both in cells and in a reporter system: hence, the authors inferred that the high potency of the molecule was mainly due to its activity on BLC-2 and that it acted both at the transcriptional (targeting DNA) and translational level (targeting RNA).

Recently, a series of three different G4-binding cNDI derivatives (compounds **56**, **37**, **57**, Table 3) [59,62,88] were tested as inhibitors of cell proliferation in glioblastoma cells [89]. Interestingly, these cNDIs showed antiproliferative activity in the nanomolar range in U251MG cells, more than 10-times better than the reference compound RHPS4 (IC_50_ 0.033 ± 0.006 and 0.460 ± 0.060 µM, respectively, Table 4) [90], while they had lower efficacy in targeting telomeres, as assessed by immunostaining and ChIP assay. This behavior, accompanied by the absence of radiosensitization in treated cells, supported the hypothesis of targets other than telomeres. Indeed, one of the molecules was able to induce BCL2 downregulation both at transcripts and protein levels and perturbation of cell cycle and apoptosis with a different mechanism of action with respect to RHPS4.

#### 4.1.3. RET

RET (REarranged during Transfection) is a proto-oncogene associated with medullary thyroid cancer containing a G4 forming sequence in its promoter [91]. A previously published molecule (compound **37**, Table 2 and Table 3) [59] was demonstrated to exert antiproliferative effect in cells (IC_50_ 1.7 ± 0.2 µM, Table 4), coupled with a remarkable downregulation of RET expression. In particular, RET expression was markedly impaired, while the effect on MYC and BCL-2 proteins was negligible. The compound efficiently recognized and stabilized the RET G4 sequence in vitro, as assessed by FRET melting (ΔT_m_ > 37.3 °C, measured by CD thermal unfolding at 10 mM K^+^), and Taq polymerase stop assay. Furthermore, in a reporter system, treatment with **37** showed a significant and dose-dependent inhibition of luciferase activity. Finally, in a mouse model of medullary thyroid cancer, treatment with this cNDI led to significant tumor growth delay and induced apoptosis [92].

#### 4.1.4. AR

The androgen receptor (AR) has a role in the development of both benign and malignant prostate cancer, even after androgen deprivation. Because castration-resistant prostate cancer (CRPC) progresses in spite of androgen deprivation, transcriptional repression of AR signaling is a rational treatment approach. 

A G4 forming region has been identified within the minimal promoter of the AR gene [93]: the three reported G4-forming sequences were characterized by means of biophysical techniques; one of them, showing the least heterogeneous NMR signal, was selected as target for molecule development. Among the seven cNDIs screened as ligands against this sequence, one of them (a tetrasubstituted cNDI, compound **58**, Table 3) showed the ability to stabilize the AR G4, even if to a lower extent than the telomeric one (ΔT_m_ 14.9 ± 0.4 °C and 25.8 ± 0.06 °C respectively, measured by FRET, Table 4). This compound also had an antiproliferative effect in the nanomolar range (IC_50_ 0.29 ± 0.02 µM, Table 4) on prostate cancer cell lines and, very interestingly, a downregulation effect on AR transcription, coupled with perturbation of some AR’s upstream transcription factors.

Recently, a library of differently functionalized cNDIs (tri- and tetrasubstituted, core extended NDIs and NDI-dyads) was screened for the ability to stabilize the AR G4s [94]. A core extended NDI (compound **59** in Table 3) was identified as the best AR G4 stabilizer by means of FRET melting assay (ΔT_m_ 31.0 ± 0.9 °C, Table 4): its affinity toward the AR G4 was in the low nanomolar range, as assessed by SPR analysis. Stabilization of the AR G4 was also confirmed by CD thermal folding analysis and Taq polymerase stop assay. The molecule had an antiproliferative effect which was greater in AR-positive (low nanomolar range, IC_50_ 0.010 ± 0.001 µM, Table 4) than in AR-negative prostate cancer cell lines. Moreover, a marked decrease in the expression levels of AR but also of its downstream regulated gene KLK3 was observed, as well as marked downregulation of genes involved in tumor cell biology, such as cell cycle regulators, apoptosis inhibitors (BCL-2), KRAS, and genes involved in the PI3K/AKT/mTOR signaling pathway. Finally, the treatment of prostate cancer cell lines with this cNDI in combination with the AR antagonist Enzalutamide (used in second line therapies) resulted in a synergistic interaction in AR-positive but not in AR-negative cell lines. 

#### 4.1.5. Multi-Targeting NDIs

As already stated, tumor malignancies are multifactorial diseases, for which the lack of selectivity could be exploited as a positive feature. Indeed, a number of NDIs targeting multiple G4 structures and exerting antiproliferative activity has been reported. 

A trisubstituted cNDI (compound **37**, Table 1 and Table 3), previously indicated to stabilize the telomeric G4, cause telomere dysfunction and downregulate telomerase activity [59], was investigated to clarify its mechanism of action by analyzing its ability to interfere with oncogene expression [88]. The compound affected the expression levels of G4-containing genes, such as TERT, MYC, BCL-2 and KIT, and preferentially bound and greatly stabilized G4s in the promoter region of MYC and BCL-2 (Table 4). Gene expression profile analyses were performed in treated cells, showing a general and cell-type dependent perturbation of expression.

A recent paper presented a trisubstituted cNDI (compound **60**, Table 3) able to affect human pancreatic ductal adenocarcinoma (PDAC) cell lines [95]. The molecule was designed by computer modeling starting from a co-crystal structure of a different cNDI in complex with an intramolecular telomeric G4 [55]: the rationally designed molecule stabilized G4s in vitro, even if less potently than the parental molecule, and was a potent antiproliferative agent, with IC_50_ in the low nanomolar range (IC_50_ 0.007 ± 0.002 µM, Table 4) in PDAC cell lines. Moreover, in a mouse xenograft model of PDAC, the cNDI showed antitumor activity. Instead of checking the activity on a selected G4 target, the authors analyzed the global genome transcriptome by means of RNA-seq, in order to identify all genes affected by the molecule: the downregulated gene set was shown to be enriched in G4 forming sequences. Moreover, cell treatment with this molecule resulted in an increase in anti-G4 antibody (BG4) foci and DNA damage recorded as double strand breaks (DSB).

### 4.2. Naphthalene Diimides Targeting Other Genes

Targeting of G4s present within the promoter of other genes can also produce an anticancer effect: it is the case of the molecules reviewed in this section, targeting G4s in the promoter of the HSP90 gene.

#### HSP90

The molecular chaperone heat shock protein 90 (HSP90) stabilizes a number of oncogenic proteins involved in tumor proliferation and malignant progression and for this reason it is considered a rational target for anticancer therapy [96]. 

Two G4 forming sequences in the promoter of HSP90 gene have been reported and characterized [97]. A series of tetrasubstituted cNDIs, positional isomers of previously published molecules, has been screened for the ability to bind and stabilize the HSP90 G4s [98]. Many of these molecules exerted a marked stabilization of the two HSP90 G4s as well as the telomeric one (ΔT_m_ 34 °C, 29 °C and 27 °C respectively, measured by FRET, Table 4); the most active of them (compound **61**, Table 3) had a cytotoxic effect on pancreatic and lung cancer cell lines in the low nanomolar range (IC_50_ 0.002 ± 0.001 µM, Table 4). Long-term telomere length studies showed no significant change in telomere length, indicating that the antiproliferative effect does not involve inhibition of telomerase. Finally, molecular dynamics studies helped the authors to understand if and how the positional isomers had any advantage in terms of G4 stabilization and inhibition of cell proliferation: in some cases, they found that more asymmetrically shaped molecules were more potent than their lead compounds, a fact that was likely due to subtle differences in the binding geometry to their targets.

## 5. Naphthalene Diimides as G4 Binders in Microorganisms

NDIs have also been employed as G4 binders in microorganisms: in particular, NDI derivatives were tested against the immunodeficiency virus 1 (HIV-1), the herpes simplex virus 1 (HSV-1), and a few parasites.

A core extended NDI derivative (compound **59**, Table 3 and Table 4) exhibited a very promising antiviral activity in the low nanomolar range against different strains of HIV-1 (IC_50_ < 25 nM) [9], combined with a very low cytotoxicity and a very promising therapeutic window. Its high affinity for the viral G4s [20] and lower affinity for the telomeric one was accounted for in its antiviral activity. The same core extended NDI was also analyzed in the context of HSV-1 infection: the molecule showed a remarkable antiviral activity (IC_50_ = 0.018 ± 0.001 nM) and was more efficient than the reference drug acyclovir, the antiviral drug of choice for the treatment of HSV-1 infections [99]. In this case, the cNDI derivative did not exhibit a net preferentiality for the viral G4s over the cellular ones, but the higher content of HSV-1 G4s during the viral cycle was indicated to play a major role in the anti-herpetic effect.

Recently, a diethylenetriamine (DETA) substituted cNDI (compound **62**, Table 5) was shown to selectively chelate Cu^2+^ and to stabilize different G4 forming sequences [100]. In particular, taking advantage of the copper-catalyzed reduction of hydrogen peroxide in the presence of ascorbate, the molecule was able to generate reactive oxygen species (ROS) that did not diffuse in solution but reacted at specific sites of the target upon G4 binding: it was the case of the specific cleavage observed against the HIV-1 LTR-III [20] and HSV-1 un2 sequences [101]. The mechanism of action was characterized by mass spectrometry (MS and MS/MS): analysis of the cleavage products indicated H1′ and H4′ abstractions by the hydroxyl radical as the main mechanism of the G4 breaks. The complex of the molecule with LTR-III was also investigated by one-dimensional ^1^H-NMR: the ligand showed a binding preference for the duplex-quadruplex junction, a peculiar feature of this sequence, reported for the first time in a naturally occurring and biological relevant G4, and recently solved by NMR [102].

Finally, a series of carbohydrate cNDI derivatives, previously evaluated as G4 binders and antitumor compounds [57], was tested against *Trypanosoma brucei*, *Leishmania major* and *Plasmodium falciparum* [103]. Two molecules (compounds **32** and **63**, Table 5), which showed antiparasitic activity against the tested species, especially against *T. brucei*, greater than the G4-stabilizing reference compound BRACO-19, TmPyP4 and pyridostatin and accumulation in the parasite nucleus and kinetoplast, suggested that the G4 targets be mainly present in these locations. This series of carbohydrate cNDI derivatives was recently implemented, modifying both the spacer between the sugar unit and the NDI core, and the substituents [104]. Most of the new members of the family displayed IC_50_ values against *T. brucei* in the sub-µM range, coupled with a notable selectivity over control cells; moreover, all the carb-NDI conjugates showed a stabilization of the telomeric and the EBR1 sequences, greater than the previously reported molecules. Again, the selective localization in the *T. brucei* nucleus and kinetoplast, targets that harbor the putative G4 forming sequences, support the hypothesis of a novel G4-mediated antiparasitic approach.

## 6. Conclusions

NDI derivatives are molecules that, by virtue of their large aromatic core, selectively bind G4s. Their properties can be largely varied by the addition of specific substituents, making them amenable to developments as attractive anticancer and antimicrobial drugs and as G4 markers in cells. 

In general, the parameters that describe the potency of G4 stabilization do not perfectly correlate with the IC_50_ anti-proliferative data. However, it is possible to envisage an overall rationalization since the best binders in general offer an outstanding biological activity. Starting from the first studied di-substituted cNDIs to the tetra-substituted one, an increase in the number of side chains corresponded to an increase in G4 stabilization. Considering that the interaction with the G4 grooves is fundamental to improve G4-ligand binding, many of the developed cNDIs bear a large positive charge on the side chains. This chemical property guarantees a greater interaction with the negative phosphate groups and good cellular permeability. However, due to the electrostatic interaction, an excess of positive charge reduces the selectivity of these NDIs towards the target, making them able to bind to other NA secondary structures as well. Enhanced G4 selectivity was thus obtained by reducing the protonable sites in the side chains and taking care not to lose the intermolecular interaction with the G4 grooves. 

In order to optimize G4 stabilization and cell entry, another crucial parameter is the length of the functionalized side chains. Based on different observations, the three-carbon atom linker guaranteed the best compromise. Nevertheless, conjugation of active transport moieties improved cellular uptake.

Moreover, the extension of the cNDIs’ aromatic core is important: this modification greatly increases the affinity towards G4s, allowing the biological activity of these derivatives to reach the low nanomolar range. 

In conclusion, the high potency and selectivity towards the NA G4 conformation make cNDI derivatives promising therapeutic agents, especially for cancer applications, where most of the G4s are involved in hallmarks of cancer. In this scenario, compounds not selective for a specific G4 could in some cases be advantageous. Conversely, for the treatment of diseases caused by infective agents, a discrete selectivity toward the target of choice would be more advisable. In this case, additional functional modifications will be needed. So far, compounds reported to have an increased selectivity for a specific G4 have added side chains that typically recognize flanking regions of the selected G4 [105]. Therefore, this may be a necessary route for the development of more selective compounds. With the compounds increasing in size, bioavailability may become an issue, which a prior accurate design of the side chains themselves could help overcome. Alternatively, a powerful screening or molecule construction towards and around the G4 target may yield small molecules with a reasonably small size that are selective for the G4 of choice [106,107]. In general, however, given that G4s demand that extensive planar moieties be optimally and selectively recognized, bioavailability of G4-ligands looks like the most impendent issue to be solved for the successful use of these compounds as therapeutic agents. 

## Figures and Tables

**Table 1:** Structures of NDIs used as reversible ligands to stabilize telomeric G4s.

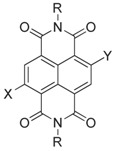				
NDI Core				
Name	R	X	Y	Ref.
**1**	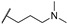	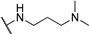	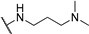	[34]
**2**	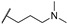	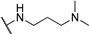	-H	[34]
**3**	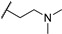	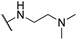	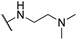	[34]
**4**	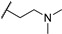	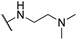	-H	[34]
**5**	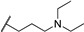	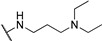	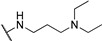	[34]
**6**	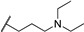	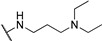	-H	[34]
**7**	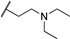	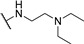	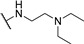	[34]
**8**	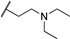	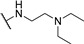	-H	[34]
**9**	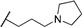	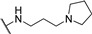	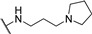	[34]
**10**	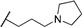	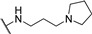	-H	[34]
**11**	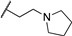	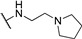	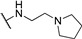	[34]
**12**	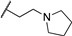	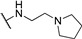	-H	[34]
**13**	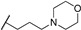	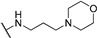	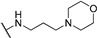	[34]
**14**	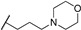	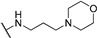	-H	[34]
**15**	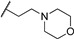	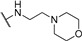	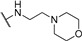	[34]
**16**	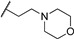	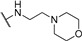	-H	[34]
**17**	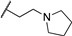	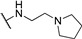	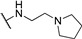	[34]
**18**	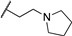	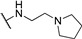	-H	[34]
**19**	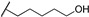	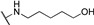	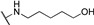	[34]
**20**	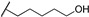	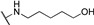	-H	[34]
**21**	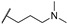	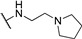	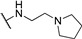	[34]
**22**	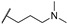	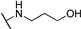	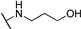	[34]
**23**	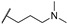	-H	-H	[34]
**24**	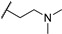	-H	-H	[34]
**25**	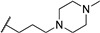	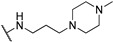	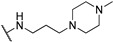	[53]
**26**	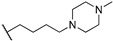	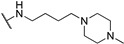	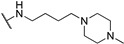	[53]
**27**	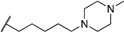	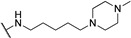	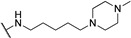	[53]
**28**	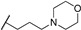	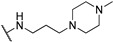	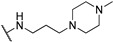	[55]
**29**	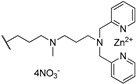	-H	-H	[56]
**30**	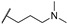	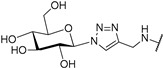	-H	[57]
**31**	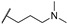	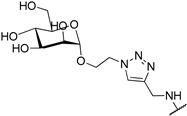	-H	[57]
**32**	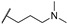	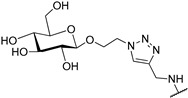	-H	[57]
**33**	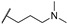	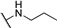	-H	[57]

**Table 2:** Structures of NDIs used as irreversible ligands to stabilize/modify telomeric G4s.

Name	R	X	Y	Ref.
**34**	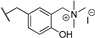	-H	-H	[58]
**35**	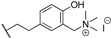	-H	-H	[58]
**36**	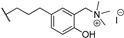	-H	-H	[58]
**37**	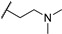	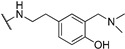	-H	[59,60,61,62]
**38**	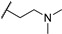	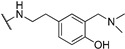	-Br	[59]
**39**	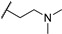	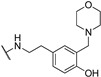	-H	[59]
**40**	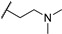	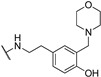	-Br	[59]
**41**	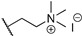	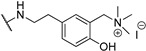	-H	[59]
**42**	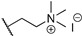	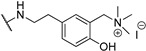	-Br	[59]
**43**	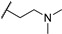	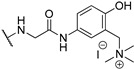	-H	[60]
**44**	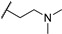	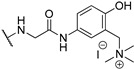	-Br	[60]
**45**	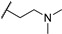	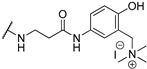	-H	[60]
**46**	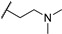	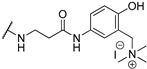	-Br	[60]
**47**	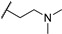	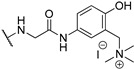	-H	[60]
**48**	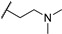	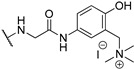	-Br	[60]
**49**	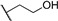	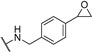	-H	[61]
**50**	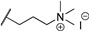	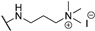	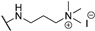	[42]

**Table 3:** Structures of NDIs used to target gene expression.

**Name**	**R**	**X**	**Y**	**Targeted Gene(s)**	**Ref.**
**11**	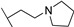	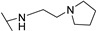	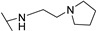	hTel, KIT, BCL-2	[76,87]
**25**	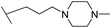	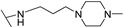	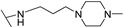	KIT, HIF1α	[77]
**55**	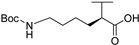	-H	-H	KIT2	[79]
**28**	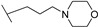	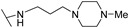	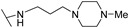	BCL-2, KRAS	[86]
**56**	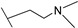	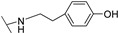	-H	TERT, BCL-2	[89]
**37**	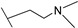	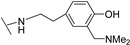	-H	TERT, BCL-2, RET, multi-targeting	[89,92]
**57**	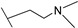	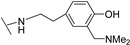	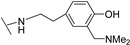	TERT, BCL-2	[89]
**60**	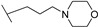	-H	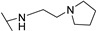	Multi-targeting	[95]
**58**	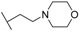	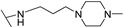	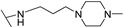	AR	[93]
**61**	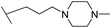	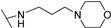	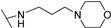	hTel, HSP90	[98]
**Name**	**Structure**		**X**	**Targeted Gene(s)**	**Ref.**
**51**	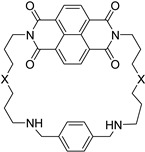		-NH-	hTel, KIT2	[78]
**52**			-NH(CH_2_)_2_NH-		
**53**			-NH(CH_2_)_3_NH-		
**54**			-NH(CH_2_)_4_NH-		
**Name**	**Structure**			**Targeted Gene(s)**	**Ref.**
**59**	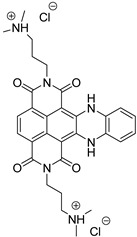			AR	[94]

**Table 4:** In vitro and cellular data of NDIs used to target gene expression.

Name	Targeted Gene(s)	ΔTm (°C) ^a^	IC_50_ (µM) ^b^	mRNA Reduction ^c^	Protein Reduction ^d^	Ref.
**11**	hTel, KIT, BCL-2	hTel 28.7/29.7, KIT-1 11.2, KIT-2 29.0, BCL-2 promoter 27.6, BCL-2 5’-UTR 10.7, dsDNA 5.7	GIST882 * 1.6, GIST48 * 0.5, GIST62 * 0.4, HGC-27 * 0.04/0.1, HT-29 * 0.03/0.04 MCF7 * 0.02/0.01	KIT ~90%, MYC 25–30%	KIT 17%, (in GIST48), KIT 90%, (in GIST882)	[76,87]
**25**	KIT, HIF1α	hTel 31.7, KIT-1 1.6, KIT-2 15.1, BCL-2 RNA 21.0, HIF1α 4.9, dsDNA 3.8				[77]
**51**	hTel, KIT2	hTel 12.6, KIT2 12.2, dsDNA 1.4	A549 * 1.0, MCF7 * 0.8, MIA-PaCa * 0.4, PANC-1* 0.4, ALT * 1.0, WI38 * 1.3			[78]
**54**	hTel, KIT2	hTel 26.8, KIT2 33.1, dsDNA 8.6	A549 * 10.4, MCF7 * > 25, MIA-PaCa * 19.3, PANC-1 * 13.6, ALT * 14.9, WI38 * > 25			[78]
**55**	KIT2	hTel 2.0, KIT-1 −1.5, KIT-2 14.6, MYC 0.1, dsDNA −2.5				[79]
**28**	BCL-2, KRAS	KRAS1 22.5, KRAS2 19.8, BCL-2 26.4			KRAS2 ~30%, BCL-2 ~40%	[86]
**56**	TERT, BCL-2		U251 ^§^ 0.180, AG01522 ^§^ 1.020		MYC slight, KIT slight, BCL-2 32.1%	[89]
**37**	TERT, BCL-2, RET, multi-targeting		U251 ^#^ 0.075, AG01522 ^#^ 0.430			[89,92]
**57**	TERT, BCL-2		U251 ^#^ 0.033, AG01522 ^#^ 0.820			[89]
**60**	Multi-targeting	hTel 11.9, HSP90A 15.7, HSP90B 12.7, KRAS21 11.0, KRAS32 9.6, BCL-2 13.3, dsDNA 0.6	A549 * 0.024, MCF7 * 0.159, MIA-PaCa2 * 0.007, PANC-1 * 0.018, WI38 * 1.190			[95]
**58**	AR	hTel 25.8, AR3 14.9, dsDNA 0.2	LNCaP ^$^ 0.42, C4-2 ^$^ 0.39, C4-2b ^$^ 0.29, PC3 ^$^ 0.68, DU145 ^$^ 0.80, PNT1a ^$^ 0.87	AR 75% (in LNCaP)	AR 4-fold (in LNCaP)	[93]
**59**	AR	AR1 20.5, AR3 31.0, dsDNA 1.0	LNCaP ^$^ 0.14, PC3 ^$^ 0.18, DU145 ^$^ 0.08, PNT1a ^$^ 0.28, 22Rv1 ^$^ 0.01	AR ~80% (in LNCaP)	AR ~55% (in LNCaP)	[93]
**61**	hTel, HSP90	hTel 34, HSP90A 36, HSP90B 34, dsDNA 9	RCC4 * 0.61, 786-O * 0.44, MCF7 * 0.014, MIA-PaCa2 * 0.050, A549 * 0.007, PANC-1 * 0.002, WI38 * 0.30			[98]

^a^ assessed by FRET melting assay on the models of the indicated sequences; ^b^ short-term antiproliferative assay at 48 h (^§^), 72 h (^$^), 96 h (*) or 120 h (^#^); ^c^ assessed by RT-PCR; ^d^ assessed by Western blot.

**Table 5:** Structures of cNDIs used to target G4s in microorganisms.

Name	Structure	Targeted Microorganism	Ref.
**59**	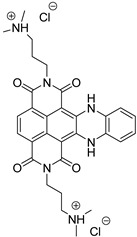	HIV-1, HSV-2	[9,20,99]
**62**	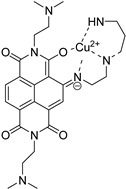	HIV-1	[100]
**32**	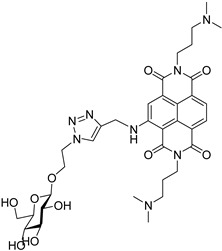	*T. brucei*, *L. major*, *P. falciparum*	[57,103,104]
**63**